# *Campylobacter jejuni* Multilocus Sequence Types in Humans, Northwest England, 2003–2004

**DOI:** 10.3201/eid1210.060048

**Published:** 2006-10

**Authors:** Will Sopwith, Andrew Birtles, Margaret Matthews, Andrew Fox, Steven Gee, Michael Painter, Martyn Regan, Qutub Syed, Eric Bolton

**Affiliations:** * Health Protection Agency (North West), Liverpool, United Kingdom;; † Regional Health Protection Agency Laboratory, Manchester, United Kingdom;; ‡ Cumbria and Lancashire Health Protection Unit, Preston, United Kingdom

**Keywords:** Campylobacter, Multilocus Sequence Typing, Epidemiology, Epidemiology, Molecular, Bacterial Typing Techniques, Great Britain, Research

## Abstract

Detailed understanding of the epidemiology of *Campylobacter* is increasingly facilitated through use of universal and reproducible techniques for accurate strain differentiation and subtyping. Multilocus sequence typing (MLST) enables discriminatory subtyping and grouping of isolate types into genetically related clonal complexes; it also has the advantage of ease of application and repeatability. Recent studies suggest that a measure of host association may be distinguishable with this system. We describe the first continuous population-based survey to investigate the potential of MLST to resolve questions of campylobacteriosis epidemiology. We demonstrate the ability of MLST to identify variations in the epidemiology of campylobacteriosis between distinct populations and describe the distribution of key subtypes of interest.

## Abstract 2

MLST can be used to describe and analyze the epidemiology of campylobacteriosis in distinct human populations.

Campylobacter has been the most commonly reported bacterial enteric pathogen causing gastrointestinal illness in England and Wales for at least the last 15 years (*1*). The main species infecting humans are Campylobacter jejuni and C. coli; these species also colonize many different animals, especially birds (*2*).

In Europe, campylobacteriosis shows a marked seasonality with a peak during the summer months (*3**,**4*), although this pattern is more marked in some countries than others (*5*). Especially sharp and annually consistent rises in incidence are reported in the United Kingdom, Greece, the Netherlands, and Denmark (*5*). A study in northwest (NW) England also indicated a consistent peak of human infection in March (*6*). Some studies have shown a coincident seasonality of infection in broiler chickens and humans in Scandinavia and contamination of retail raw chicken and infection in humans in the United Kingdom and suggest a common environmental trigger (*2**,**7**,**8*). In a recent study of the influence of rainfall, sunshine, and temperature on seasonality in different regions of England and Wales, increasing incidence of campylobacteriosis was most strongly correlated with increases in air temperature (*9*).

Studies in northern Europe have attempted to identify environmental reservoirs of infection in water sources and livestock that could explain the seasonality of human infection. These studies have demonstrated campylobacter carriage rates peaking in late spring and summer in broiler chicken flocks (*10**,**11*) and dairy cattle (*12*) but more constant infection in lambs and beef cattle (*12**,**13*). Campylobacter has been successfully isolated and cultured from surface water in Finland (*14**,**15*), Italy (*16*), and NW England (*17*), and sporadic campylobacteriosis (illness not associated with an outbreak) has been linked with exposure to untreated water in Scandinavia (*18**,**19*). C. jejuni has also been isolated from a wide range of animal and environmental samples in a rural area in NW England, which suggests a potential environmental risk for exposure (*20*).

Molecular subtyping methods including pulsed-field gel electrophoresis (PFGE) (*21*), fluorescent amplified fragment length polymorphisms (*22*), and multilocus sequence typing (MLST) (*23*) have been applied to C. jejuni to overcome the problems associated with traditional phenotypic methods. Although PFGE has been accurately and reproducibly used for several years to investigate disease clusters (*24*), an advantage of MLST is its ability to sequence isolate types and thus group them into genetically related clonal complexes; this is especially useful for integrating newly identified sequence types. MLST has also provided conceptual advances in understanding the population biology and epidemiology of C. jejuni (*25*). Previous studies that used phenotypic strain characterization methods failed to establish source/host associations for particular phenotypes (*26*), but recent studies with MLST, including a study in NW England, suggest that a measure of host association may be distinguishable when this system is used (*27*).

No continuous population-based survey has been performed to investigate the potential of MLST to answer the unresolved questions of campylobacteriosis epidemiology, particularly the drivers of the seasonal peak in the United Kingdom. This article is the first report of a 3-year study that used MLST to investigate campylobacteriosis in NW England in 2 defined human populations, 1 small town–based and rural and l metropolitan and suburban.

## Methods

### Study Population

**Figure 1:**
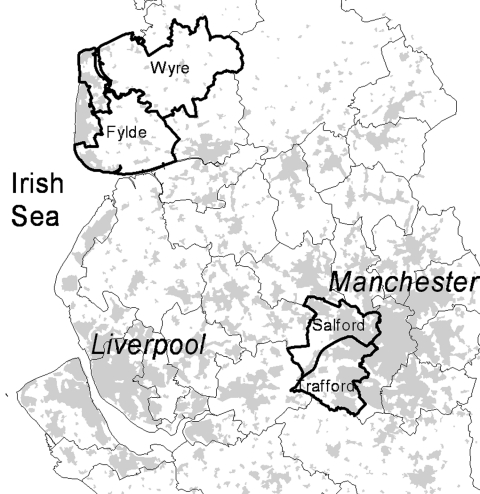
Location of the 4 local authorities constituting the study area in Northwest England. The populations covered (outlined areas) were Fylde and Wyre (in Lancashire) and Salford and Trafford (within the metropolitan area of Greater Manchester). Gray areas indicate approximate location of built-up areas.

The study population was defined as all persons with confirmed Campylobacter from April 2003 to March 2004 reported by residents in 4 local authorities (government administrative boundaries) in NW England (Figure 1). Wyre (population 106,826) and Fylde (74,032) local authorities adjoin geographically in the county of Lancashire and largely consist of rural and small town–based populations. For the purposes of this study, this area is referred to as rural. Salford (216,178) and Trafford (209,760) local authorities also adjoin geographically within the conurbation of Greater Manchester and are predominantly a mix of metropolitan and suburban districts. This area is referred to as suburban in this study. The 2 areas are ≈50 km apart and share some of the same drinking water sources but are environmentally located in different water catchment areas.

### Data Collection

Confirmed cases of campylobacteriosis (according to the UK National Standard Method for diagnosis [28]) are routinely reported to the NW Health Protection Agency (HPA) surveillance system by local National Health Service laboratories. Case-patients were identified as residents in the study area through available geographic information or by patient names, when geographic information was not available. Reports included basic demographic information such as age, sex, and date of disease onset. Where date of onset was not available, date of report was used as a proxy. Travel overseas was not well recorded in these data.

Positive isolates of Campylobacter from patients resident in the study area were sent by the main diagnostic laboratories to the NW HPA Laboratory in Manchester for sequence typing. Case-patients were determined to be residents in the study area by using the methods described above.

### Campylobacter Speciation

C. jejuni isolates were spread onto Colombia blood agar (Oxoid CM331, Unipath, UK) containing 5% defibrinated horse blood and incubated at 37°C in anaerobic jars (Don Whitley Scientific, Shipley, UK) under microaerobic conditions (5% CO2, 5% O2, 3% H2, 87% N2). DNA was extracted (10% dilutions) and tested for C. jejuni or C. coli by using a previously described Taqman assay (*29*) with primers and probes for the genes ceuE (for C. coli) and mapA (for C. jejuni).

### Sequence Typing of C. jejuni Isolates

MLST was performed as described (*23*). The amplification reactions were performed in a 50-μL volume containing ≈1 μL C. jejuni chromosomal DNA (10 ng/μL), 5 μmL of each primer (10 pmol/μL), 10 μL of 1 mmol/L deoxynucleoside triphosphates (Roche, Welwyn Garden City, UK), 5 μL 10× PCR buffer (Qiagen, Crawley, UK), and 0.25 U Taq DNA polymerase (Qiagen). The thermal cycling conditions were as follows: initial denaturation at 94°C for 5 min, 40 cycles of denaturation at 94°C for 2 min; primer annealing at 50°C for 1 min; and extension at 72°C for 1 min with a final elongation step at 72°C for 10 min. Thermal cycling was conducted with an MJ PTC 200 thermal cycler. Amplicons were detected on a 1.5% ethidium bromide agarose gel and purified by using a UniFilter Multiscreen PCR cleanup plate (Whatman, Brentford, UK) according to the manufacturer's instructions. Sequencing reactions were conducted in 10 μL of 1/4 reaction volumes containing 2 μL purified DNA, 0.5 μL primer (10 pmol/μL), 1 μL sequencing buffer (Genetix, New Milton, UK), 2 μL DTCS Quick Start Master Mix (Beckman Coulter, Fullerton, CA, USA), and 4.5 μL molecular grade water. Thermal cycling conditions for sequencing reactions and ethanol cleanup of sequenced products were set up according to the manufacturer's instructions (Beckman Coulter). The products were analyzed on a Beckman Coulter CEQ 8000 automated DNA sequencer (Beckman Coulter). All sequence assemblage and editing were performed with Sequencher 4.0 software (GeneCodes Corporation, Ann Arbor, MI, USA).

### MLST Allele and ST Assignment

MLST alleles, sequence types (STs), and clonal complexes were assigned by using the Campylobacter PubMLST database (*30*). Sequences were submitted for allele designation as appropriate.

### Statistical Analysis

Incidence rates quoted were calculated per 100,000 population by using the relevant 2003 annual population estimate for each local authority area, purchased from the UK Office for National Statistics. Incidence ratios, associated 95% confidence intervals (CIs), and p values were calculated by using StatsDirect statistical software (http://www.statsdirect.com/), which used Fisher exact test to analyze the difference between 2 crude rates. A mid-p approach to Fisher exact test was also used to test the significance of observed differences in proportions (by using StatsDirect). The level of statistical significance chosen for all analyses was p<0.05.

## Results

### Seasonal Incidence of Reported Disease

**Figure 2:**
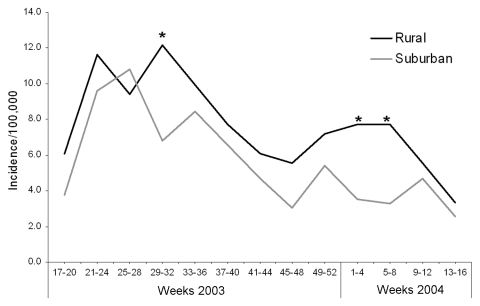
Seasonality of human cases of campylobacteriosis reported in the first 12 months of the study period in patients residing in Fylde and Wyre (rural) and Salford and Trafford (suburban). To allow comparison between the areas, the number of cases reported to the North West Health Protection Agency surveillance system during 4-week intervals were converted to incidence by using estimates of the annual population for each local authority. The periods at which the incidence differed with marginal statistical significance are indicated with an asterisk: weeks 29–32, incidence ratio (IR) 1.79, 95% confidence intervals (CI) 0.98–3.22 (p = 0.05); weeks 1–4, IR 2.20, 95% CI 0.98–4.88 (p = 0.04); weeks 5–8, IR 2.36, 95% CI 1.04–5.33 (p = 0.02).

During the first 12 months of the study period (April 2003–March 2004), 493 cases of laboratory-confirmed Campylobacter sp. were reported through the NW surveillance system (Table 1) from residents of Fylde, Wyre, Salford, and Trafford local authorities (Figure 1). This corresponded to approximate annual incidences of 100/100,000 for the area encompassing Fylde and Wyre (rural area) and 73.3/100,000 for the area encompassing Salford and Trafford (suburban area); this difference was significant (p<0.001). The incidence for the whole region of NW England in this period was 69.8/100,000 (data not shown). A similar seasonal pattern of cases was seen in each of the 2 areas in the study, with a large increase in reported cases between weeks 17 to 20 and weeks 21 to 24 (May/June), an elevated incidence through the summer months (weeks 21–36) that declines between weeks 37 and 48 (September to November) to the baseline level of incidence seen in weeks 17–20 (Figure 2). Incidence appeared in increase during the Christmas holiday period (weeks 49–52); this increase was sustained in the rural area until week 8 (February). With the exception of one 4-week period (weeks 25–28), the monthly incidence of campylobacteriosis reported was higher in the rural area, but this finding was only statistically significant during weeks 29–32 in 2003 and weeks 1–8 in 2004.

**Table 1:** Distribution of *Campylobacter jejuni* multilocus sequence typing clonal complexes by study area, April 2003 to March 2004*

Clonal complex	Fylde	Wyre	Salford	Trafford	Total	% of all typed
ST-21	15	21	22	44	102	28.7
ST-45	11	10	7	7	35	9.8
UA	6	6	6	16	34	9.6
ST-257	5	4	9	10	28	7.9
ST-443	1	5	6	9	21	5.9
ST-48	3	3	2	11	19	5.3
ST-206	6	2	3	7	18	5.1
ST-353	3	1	1	8	13	3.7
ST-22	2	0	2	3	7	2.0
ST-49	0	2	2	3	7	2.0
ST-42	1	0	2	3	6	1.7
ST-354	1	0	0	4	5	1.4
ST-61	2	1	1	1	5	1.4
ST-283	0	1	0	3	4	1.1
ST-52	1	0	2	1	4	1.1
ST-573	1	2	0	1	4	1.1
ST-658	1	0	2	1	4	1.1
ST-403	0	0	0	3	3	0.8
ST-508	1	0	0	2	3	0.8
ST-460	0	0	2	0	2	0.6
ST-177	1	0	0	0	1	0.3
ST-362	0	0	1	0	1	0.3
*C. coli*	5	11	4	10	30	8.4
No typing	22	24	39	52	137†	
Total	88	93	113	199	493	

*Including UA (new sequence types as yet unassigned to a clonal complex). Data show the number of human isolates per local authority and the percentage of all typed isolates attributed to each complex. Numbers of *C. coli* isolates are also shown and included in the denominator of "all typed cases." Isolates with "no typing" represent reports of campylobacteriosis to the surveillance system that do not have a corresponding typed isolate. †Not included in the "all typed cases" denominator.

### Sequence Typing of Isolates

Of 493 cases reported, 388 (79%) laboratory specimens were obtained for typing. A proportion of the other cases may not have been identified at the point of diagnosis as study isolates because incomplete demographic information was supplied with the sample. Of the specimens submitted for typing, 30 were C. coli, and 32 did not yield a culture, which left 326 isolates (66% of reported cases) of C. jejuni typed. From these isolates, 93 distinct MLST sequence types of C. jejuni were identified and assigned to 21 clonal complexes, with 20 remaining unassigned until further identification of types enables the designation of new complexes. The most common clonal complex isolated was ST-21 (102 cases, 28.7% of all typed cases, including C. jejuni and C. coli), and this complex was almost 3 times more common than the next complex ST-45 (35 cases, 9.8% of all typed cases) (Table 1). Almost 10% of typed cases were unassigned to clonal complexes at time of writing.

### Geographic Distribution of Sequence Types

The geographic distribution of clonal complexes across the study area varied. Of the 10 most commonly reported clonal complexes (including ones not yet assigned), several were reported with higher incidence in the rural area, although greater numbers are required to give sufficient power to test the significance of these differences in many groups (Table 2). The greatest variation was seen among cases with clonal complexes ST-45 (incidence ratio [IR] 3.53, 95% CI 1.71–7.51) and ST-206 (IR = 1.88, 95%CI 0.65–5.30), although ST-45 was the only complex with a significantly (p<0.001) higher incidence in the rural area. Although not sequence typed, the incidence of C. coli (IR 2.69, 95%CI 1.23–5.95) was also significantly higher in the rural area (p<0.01).

**Table 2:** Comparative distributions of the most common MLST clonal complexes by study area, April 2003 to March 2004*

Clonal complex	Isolates		Incidence/100,000		Incidence ratio	95% confidence intervals	p value
	Rural	Suburban	Rural	Suburban			
ST–21	36	66	19.91	15.50	1.28	0.83–1.96	–
**ST–45**	**21**	**14**	**11.61**	**3.29**	**3.53**	**1.71–7.51**	**<0.001**
UA	12	22	6.64	5.17	1.28	0.58–2.71	–
ST–257	9	19	4.98	4.46	1.12	0.44–2.59	–
ST–443	6	15	3.32	3.52	1.01	0.32–2.80	–
ST–48	6	13	3.32	3.05	1.09	0.34–3.07	–
ST–206	8	10	4.42	2.35	1.88	0.65–5.30	–
ST–353	4	9	2.21	2.11	1.05	0.24–3.75	–
ST–22	2	5	1.11	1.17	0.94	0.09–5.75	–
ST–49	2	5	1.11	1.17	0.94	0.09–5.75	–
C. coli	**16**	**14**	**8.85**	**3.29**	**2.69**	**1.23–5.95**	**<0.01**
All cases†	**181**	**312**	**100.08**	**73.25**	**1.37**	**1.13–1.65**	**<0.001**

*Including UA (new sequence types as yet unassigned to a clonal complex), *Campylobacter coli* isolates, and all cases of campylobacteriosis reported. Annual estimated incidence was calculated for each clonal complex and incidence ratios were calculated for rural incidence/suburban incidence, including confidence intervals. Those data with a statistically significant difference between the study areas are shown in **boldface** type (level of significance p<0.05). MLST, multilocus sequence typing. †Refers to the entire dataset for reference, i.e., those shown in this table plus all others.

### Temporal Distribution of Sequence Types

**Figure 3:**
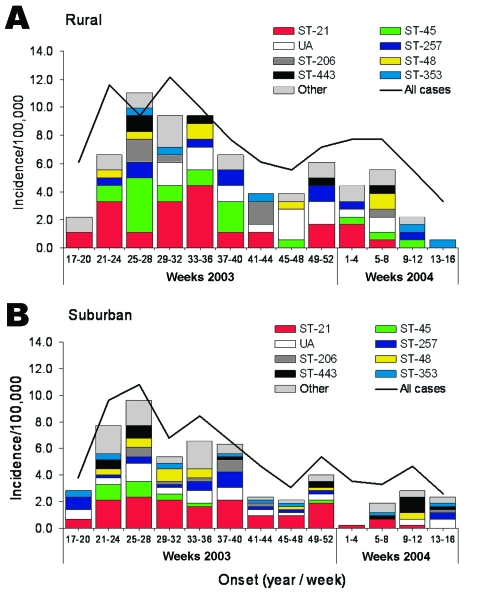
Seasonal distribution of multilocus sequence typing clonal complex in human cases of campylobacteriosis reported in the first 12 months of the study period, by residence in A) Fylde and Wyre (rural) and B) Salford and Trafford (suburban). The number of typed isolates reported during 4-week intervals was converted to incidence by using annual population estimates. The 8 most commonly reported complexes are distinguished, with cases from all other complexes presented as "other." The incidence of "all cases reported" (typed and untyped) is presented for reference (solid line).

Temporal distribution of different clonal complexes in each of the study areas also varied through the first year of the study. Cases of clonal complex ST-45 were most often reported in the rural area during weeks 25–28, although cases were reported continuously throughout the summer months (weeks 21–40, May to September, Figure 3). The proportion of cases reported in weeks 25 to 28 that were typed ST-45 was significantly higher that reported in the annual rural dataset (proportion difference 0.188, exact mid-p = 0.038) (data not shown). In the suburban area, clonal complex ST-45 was most often reported during weeks 21–28 (May to July), and the temporal distribution appeared reduced. The only period in which the difference in ST-45 incidence between the areas approached significance was weeks 25–28 (IR 3.3, 95% CI 0.9–13.17, p = 0.052) (data not shown). Clonal complex ST-45 was not reported before week 21 in either area.

Cases of clonal complex ST-21 were reported more or less throughout the 12-month period in both areas, but the incidence fluctuated far less in the suburban area. Almost 50% of the cases reported in the suburban area in weeks 49 to 52 were clonal complex ST-21 compared with 30% in the annual suburban dataset, but this difference was not significant (proportion difference 0.176, exact mid-p = 0.113) (data not shown).

Clonal complex ST-206 was most often reported in the rural area in weeks 25 to 28, and more remarkably in weeks 41 to 44, when the proportion of cases typed as ST-206 was significantly higher than in the annual dataset (proportion difference 0.367, exact mid-p = 0.006) (data not shown). Cases of clonal complex ST-206 were consistently reported from weeks 25 through 44 (June to October) in the suburban area, but the proportion of ST-206 cases in weeks 37 to 40 was significantly higher than in the annual dataset (proportion difference 0.101, exact mid-p = 0.035).

### Age Distribution of Sequence Types

Analysis of age-specific incidence by clonal complex was hampered by low numbers of cases; only data for the 6 most commonly reported complexes are shown (Table 3). Overall, the higher incidence of campylobacteriosis described in the rural area was also evident in each of the age bands analyzed, although the difference in 0- to 14-year-old patients was only marginally significant (IR 1.72, 95% CI 0.91–3.18). Among cases in the most commonly reported clonal complex, ST-21, incidence among younger case-patients (0- to 14-year-olds) was higher in the suburban area, but for all other age groups, the incidence was higher in the rural area. None of these differences were statistically significant. The only significant difference was a higher incidence of ST-45 among those >55 years of age in the rural area compared with those >55 years in the suburban area.

**Table 3:** Comparative age distributions of the most common MLST clonal complexes by study area (first year of the study)*

Clonal complex	Age group	Isolates		Incidence/100,000		Incidence ratio	95% confidence intervals	p value
		Rural	Suburban	Rural	Suburban			
ST–21	0–14	2	14	6.65	17.68	0.38	0.04–1.64	–
	15–34	9	24	24.27	21.02	1.15	0.47–2.57	–
	35–54	12	18	24.38	15.14	1.61	0.71–3.53	–
	>55	13	13	20.17	11.43	1.76	0.75–4.13	–
ST–45	0–14	3	1	9.97	1.26	7.89	0.63–414.40	–
	15–34	1	4	2.70	3.50	0.77	0.02–7.78	–
	35–54	7	6	14.22	5.05	2.82	0.81–10.15	–
	> **55**	**10**	**3**	**15.51**	**2.64**	**5.88**	**1.51–33.24**	**<0.01**
ST–257	0–14	2	1	6.65	1.26	5.26	0.27–310.48	–
	15–34	1	8	2.70	7.01	0.38	0.01–2.87	–
	35–54	3	8	6.09	6.73	0.91	0.15–3.77	–
	> **55**	4	4	6.21	3.52	1.76	0.33–9.47	–
ST–443	0–14	1	0	3.32	0.00	–	–	–
	15–34	3	5	8.09	4.38	1.85	0.29–9.50	–
	35–54	0	6	0.00	5.05	–	–	–
	> **55**	1	6	1.55	5.28	0.29	0.01–2.42	–
ST–48	0–14	1	2	3.32	2.53	1.32	0.02–25.27	–
	15–34	5	7	13.48	6.13	2.20	0.55–8.05	–
	35–54	0	4	0.00	3.36	–	–	–
	> **55**	1	2	1.55	1.76	0.88	0.01–16.94	–
ST–206	0–14	0	0	0.00	0.00	–	–	–
	15–34	1	6	2.70	5.26	0.51	0.01–4.23	–
	35–54	5	5	10.16	4.21	2.42	0.56–10.49	–
	> **55**	2	1	3.10	0.88	3.53	0.18–208.11	–
All cases	0–14	19	29	63.13	36.62	1.72	0.91–3.18	–
	**15–34**	**48**	**87**	**129.45**	**76.20**	**1.70**	**1.17–2.44**	**<0.01**
	**35–54**	**54**	**87**	**109.71**	**73.18**	**1.50**	**1.05–2.13**	**0.02**
	> **55**	**57**	**66**	**88.43**	**58.05**	**1.52**	**1.05–2.20**	**0.02**

*Including all cases of campylobacteriosis reported for which age was available. Annual estimated incidences in 4 age groups were calculated for each clonal complex using estimates of the age-specific annual population for each local authority. Incidence ratios were calculated for rural incidence/suburban incidence and 95% confidence intervals calculated. Those data with a statistically significant difference between the study areas are shown in **boldface** type (level of significance p<0.05). MLST, multilocus sequence typing.

## Discussion

We described some characteristics of human infection with Campylobacter in 2 environmentally distinct areas of NW England and some preliminary results of a study that used MLST to better define the epidemiology of campylobacteriosis within a distinct population. MLST identified possible variations in the epidemiology of campylobacteriosis between populations. However, our sample sizes are small, and the role of chance in the associations described cannot be excluded without further analysis of a larger dataset.

The seasonality of incidence in the 2 study areas is broadly similar to that previously described (*3**,**6*) with a sharp rise in cases around weeks 21 to 24 (May and June) that is sustained through the summer months. Throughout the first year of the study, incidence was higher in the rural area of Fylde and Wyre than in the suburban area of Salford and Trafford; this difference was most significant in the first 8 weeks of 2004. This difference in incidence was seen for all analyzed age groups, although it was not statistically significant among 0- to 14-year-olds. These observations may indicate increased exposure of the more rural population to sources of Campylobacter and true differences in distribution season between the 2 areas, perhaps indicating distinct transmission routes. However, a variety of other factors likely influenced these observations, including differences in healthcare-seeking behavior between the populations and differences in frequency of travel abroad. Although some adjustments have been made for the underlying population structure of each study area (the use of age-specific 4-weekly incidence), estimates of population do not take into account the seasonal movement of persons, such as students (Salford has a large student population) and age-related travel (*31**,**32*). For instance, the winter rise in incidence in the rural setting may be due, in part, to winter vacations taken by those with the flexibility and finances to travel abroad out of season (generally the older generation, who are also more represented in the rural area of this study). The exclusion of travel-related cases will be key in further exploring some of these trends, and matching more detailed epidemiologic information from case questionnaires will facilitate this as the study progresses. Subsequent years of data analysis will also clarify any true differences in incidence between the populations.

Sequence typing isolates collected throughout the first 12 months of the study showed a wide range of identified types, with many represented only by single cases and relatively few identified in significant numbers. Several new types were described in this study and await assignment to clonal complexes. Almost 30% of typed isolates from the study area align with the largest clonal complex so far defined, complex ST-21 (*23**,**30*), and previously reported isolates from this clonal complex originate from a wide variety of sources other than human cases, including cattle, chicken, milk, sand, and water (*23*). Most cases arising from a large waterborne outbreak of campylobacteriosis in Walkerton, Ontario, that originated from infected cattle (*33*) were later identified as clonal complex ST-21 (*34*), which suggests that this clonal complex can be associated with environmental and foodborne transmission. Study of a farm ecosystem in NW England demonstrated that most clonal complex ST-21 isolates came from livestock, especially cattle (*27*). Isolates of clonal complex ST-21 have also been described in cloacal and excreta samples from broiler chickens (*35*) and from a variety of farmyard isolates, including sheep and wild birds (*36*).

Isolates from the next most represented clonal complex in the study area, complex ST-45, have previously been reported largely from humans and chicken (*23**,**37*) and were almost exclusively reported in broiler chicken and turkey chicks in a study sampling various livestock and wild birds in NW England (*36*). However, complex ST-45 was more frequently identified in wildlife than in livestock in the farm ecosystem study above (*27*) (although the ecosystem studied did not include poultry).

Initial data from this current study suggest that ST-45 complex is more frequently identified in the rural component of the study population than in the metropolitan component, and particularly in those >55 years of age. This finding raises the possibility that this complex may be associated with an environmental transmission pathway, a specific set of behavioral traits, or both. This hypothesis is supported by the identification of this clonal complex in wildlife isolates of Campylobacter (*27*), which suggests a widespread distribution maintained in the environment. Cases of C. coli in this study have a similar distribution, and in previous studies this species has been the most dominant one isolated from surface waters (*20**,**38*). The seasonal clustering of ST-45 complex human isolates (compared with ST-21 complex) in summer months in the study populations may also indicate an environmental source. However, seasonal carriage rates in broiler flocks in other north European settings (*10**,**11*) correlate with the seasonality of ST-45 complex in this population, in which infection of beef cattle (*13*) correlates more closely to the seasonality of ST-21 complex. Given the apparent host preferences (not exclusively) of these 2 complexes, the seasonality of human complexes in this study may only reflect Campylobacter distribution in food animals rather than a common environmental source, as suggested by previous studies (*2**,**7**,**8*).

No single sequence type was associated with the late spring seasonal rise in human campylobacteriosis, a finding consistent with that of a previous study in England that used serotyping (*26*). However, we have shown complex ST-45 to be significantly more prevalent during summer months in rural than in suburban areas. In addition, some evidence exists that a Christmas rise in infection in the suburban population may in part be mediated by cases of ST-21. Eating out at a restaurant is a recognized risk factor for campylobacteriosis (*32**,**39*), and the Christmas season involves a variety of public as well as private parties in the United Kingdom. Identifying specific sequence types associated with such seasonal activity may help clarify the role of subpopulations of Campylobacter in human epidemiology. Some evidence also suggests that MLST is able to distinguish temporal clusters of campylobacteriosis such as for ST-206, which is significantly overrepresented in weeks 41 to 44 in the rural area.

As this 3-year study progresses, we will match MLST complexes with common epidemiologic exposures through the use of case questionnaire data. The ease of use of the technique and its repeatability in a variety of laboratories are distinct advantages, and the increased use of MLST will enable valuable interlaboratory comparisons of types from similar population-based studies. We have demonstrated the ability to improve linking of apparently sporadic cases encountered in routine surveillance by assigning isolates to sequence type complexes. We believe that MLST will be a valuable tool in testing the significance of suspected epidemiologic exposures in human campylobacteriosis and thus support improved surveillance and development of effective interventions.

## References

[1] Public Health Labortory Services (PHLS). Gastrointestinal infections. PHLS. 1998 Annual review of communicable diseases. London: PHLS publications; 2000. p. 79–88.

[2] Altekruse SF, Hunt JM, Tollefson LK, Madden JM. Food and animal sources of human Campylobacter jejuni infection. J Am Vet Med Assoc. 1994;204:57–61. 8125823

[3] Nylen G, Dunstan F, Palmer SR, Andersson Y, Bager F, Cowden J, The seasonal distribution of campylobacter infection in nine European countries and New Zealand. Epidemiol Infect. 2002;128:383–90. 10.1017/S0950268802006830 12113481 PMC2869833

[4] Altekruse SF, Stern NJ, Fields PI, Swerdlow DL. Campylobacter jejuni—an emerging foodborne pathogen. Emerg Infect Dis. 1999;5:28–35. 10.3201/eid0501.990104 10081669 PMC2627687

[5] Kovats RS, Edwards SJ, Charron D, Cowden J, D'Souza RM, Ebi KL, Climate variability and campylobacter infection: an international study. Int J Biometeorol. 2005;49:207–14. 10.1007/s00484-004-0241-3 15565278

[6] Sopwith W, Ashton M, Frost JA, Tocque K, O'Brien S, Regan M, Enhanced surveillance of campylobacter infection in the North West of England 1997–1999. J Infect. 2003;46:35–45. 10.1053/jinf.2002.1072 12504607

[7] Wilson IG. Salmonella and Campylobacter contamination of raw retail chickens from different producers: a six year survey. Epidemiol Infect. 2002;129:635–45. 10.1017/S0950268802007665 12558349 PMC2869928

[8] Meldrum RJ, Griffiths JK, Smith RM, Evans MR. The seasonality of human campylobacter infection and Campylobacter isolates from fresh, retail chicken in Wales. Epidemiol Infect. 2005;133:49–52. 10.1017/S0950268804003188 15724710 PMC2870221

[9] Louis VR, Gillespie IA, O'Brien SJ, Russek-Cohen E, Pearson AD, Colwell RR. Temperature-driven Campylobacter seasonality in England and Wales. Appl Environ Microbiol. 2005;71:85–92. 10.1128/AEM.71.1.85-92.2005 15640174 PMC544220

[10] Jacobs-Reitsma WF, Bolder NM, Mulder RW. Cecal carriage of Campylobacter and Salmonella in Dutch broiler flocks at slaughter: a one-year study. Poult Sci. 1994;73:1260–6. 7971669 10.3382/ps.0731260

[11] Kapperud G, Skjerve E, Vik L, Hauge K, Lysaker A, Aalmen I, Epidemiological investigation of risk factors for campylobacter colonization in Norwegian broiler flocks. Epidemiol Infect. 1993;111:245–55. 10.1017/S0950268800056958 8405152 PMC2271384

[12] Stanley KN, Wallace JS, Currie JE, Diggle PJ, Jones K. The seasonal variation of thermophilic campylobacters in beef cattle, dairy cattle and calves. J Appl Microbiol. 1998;85:472–80. 10.1046/j.1365-2672.1998.853511.x 9750278

[13] Stanley KN, Wallace JS, Currie JE, Diggle PJ, Jones K. Seasonal variation of thermophilic campylobacters in lambs at slaughter. J Appl Microbiol. 1998;84:1111–6. 10.1046/j.1365-2672.1998.00450.x 9717297

[14] Aho M, Kurki M, Rautelin H, Kosunen TU. Waterborne outbreak of Campylobacter enteritis after outdoors infantry drill in Utti, Finland. Epidemiol Infect. 1989;103:133–41. 10.1017/S0950268800030430 2776848 PMC2249494

[15] Hanninen ML, Niskanen M, Korhonen L. Water as a reservoir for Campylobacter jejuni infection in cows studied by serotyping and pulsed-field gel electrophoresis (PFGE). Zentralblatt Fuer Veterinaermedizin Reihe B. 1998;45:37–42. 9529995 10.1111/j.1439-0450.1998.tb00764.x

[16] Baffone W, Bruscolini F, Pianetti A, Biffi MR, Brandi G, Salvaggio L, Diffusion of thermophilic Campylobacter in the Pesaro-Urbino area (Italy) from 1985 to 1992. Eur J Epidemiol. 1995;11:83–6. 10.1007/BF01719950 7489778

[17] Jones K, Betaieb M, Telford DR. Thermophilic campylobacters in surface waters around Lancaster, UK: negative correlation with Campylobacter infections in the community. J Appl Bacteriol. 1990;69:758–64. 10.1111/j.1365-2672.1990.tb01573.x 2276990

[18] Schonberg-Norio D, Takkinen J, Hanninen ML, Katila ML, Kaukoranta SS, Mattila L, Swimming and Campylobacter infections. Emerg Infect Dis. 2004;10:1474–7. 15496253 10.3201/eid1008.030924 PMC3320392

[19] Carrique-Mas J, Andersson Y, Hjertqvist M, Svensson A, Torner A, Giesecke J. Risk factors for domestic sporadic campylobacteriosis among young children in Sweden. Scand J Infect Dis. 2005;37:101–10. 10.1080/00365540510027165 15764201

[20] Brown PE, Christensen OF, Clough HE, Diggle PJ, Hart CA, Hazel S, Frequency and spatial distribution of environmental Campylobacter spp. Appl Environ Microbiol. 2004;70:6501–11. 10.1128/AEM.70.11.6501-6511.2004 15528512 PMC525266

[21] Olsen SJ, Hansen GR, Bartlett L, Fitzgerald C, Sonder A, Manjrekar R, An outbreak of Campylobacter jejuni infections associated with food handler contamination: the use of pulsed-field gel electrophoresis. J Infect Dis. 2001;183:164–7. 10.1086/317657 11078485

[22] Champion OL, Best EL, Frost JA. Comparison of pulsed-field gel electrophoresis and amplified fragment length polymorphism techniques for investigating outbreaks of enteritis due to campylobacters. J Clin Microbiol. 2002;40:2263–5. 10.1128/JCM.40.6.2263-2265.2002 12037105 PMC130728

[23] Dingle KE, Colles FM, Wareing DR, Ure R, Fox AJ, Bolton FE, Multilocus sequence typing system for Campylobacter jejuni. J Clin Microbiol. 2001;39:14–23. 10.1128/JCM.39.1.14-23.2001 11136741 PMC87672

[24] Gerner-Smidt P, Hise K, Kincaid J, Hunter S, Rolando S, Hyytia-Trees E, PulseNet USA: a five-year update. Foodborne Pathog Dis. 2006;3:9–19. 10.1089/fpd.2006.3.9 16602975

[25] Dingle KE, Colles FM, Ure R, Wagenaar JA, Duim B, Bolton FJ, Molecular characterization of Campylobacter jejuni clones: a basis for epidemiologic investigation. Emerg Infect Dis. 2002;8:949–55. 12194772 10.3201/eid0809.02-0122 PMC2732546

[26] Owen RJ, Slater E, Telford D, Donovan T, Barnham M. Subtypes of Campylobacter jejuni from sporadic cases of diarrhoeal disease at different locations in England are highly diverse. Eur J Epidemiol. 1997;13:837–40. 10.1023/A:1007497005152 9384275

[27] French N, Barrigas M, Brown P, Ribiero P, Williams N, Leatherbarrow H, Spatial epidemiology and natural population structure of Campylobacter jejuni colonizing a farmland ecosystem. Environ Microbiol. 2005;7:1116–26. 10.1111/j.1462-2920.2005.00782.x 16011749

[28] Health Protection Agency Standards Unit. Identification of Campylobacter species, BSOP ID 23. [cited 2005 Nov]. Available from http://www.hpa-standardmethods.org.uk/documents/bsopid/pdf/bsopid23.pdf

[29] Best EL, Powell EJ, Swift C, Grant KA, Frost JA. Applicability of a rapid duplex real-time PCR assay for speciation of Campylobacter jejuni and Campylobacter coli directly from culture plates. FEMS Microbiol Lett. 2003;229:237–41. 10.1016/S0378-1097(03)00845-0 14680705

[30] Jolley K, Chan M-S. Campylobacter jejuni and Campylobacter coli MLST Home Page [cited 2005 Nov]. Available from http://pubmlst.org/campylobacter/

[31] Campylobacter Sentinel Surveillance Scheme Collaborators. Foreign and domestic travel and the risk of Campylobacter infection: results from a population-based sentinel surveillance scheme. J Travel Med. 2003;10:136–8. 12650661

[32] Rodrigues LC, Cowden JM, Wheeler JG, Sethi D, Wall PG, Cumberland P, The study of infectious intestinal disease in England: risk factors for cases of infectious intestinal disease with Campylobacter jejuni infection. Epidemiol Infect. 2001;127:185–93. 10.1017/S0950268801006057 11693495 PMC2869737

[33] Clark CG, Price L, Ahmed R, Woodward DL, Melito PL, Rodgers FG, Characterization of waterborne outbreak-associated Campylobacter jejuni, Walkerton, Ontario. Emerg Infect Dis. 2003;9:1232–41. 14609457 10.3201/eid0910.020584 PMC3033067

[34] Clark CG, Bryden L, Cuff WR, Johnson PL, Jamieson F, Ciebin B, Use of the oxford multilocus sequence typing protocol and sequencing of the flagellin short variable region to characterize isolates from a large outbreak of waterborne Campylobacter sp. strains in Walkerton, Ontario, Canada. J Clin Microbiol. 2005;43:2080–91. 10.1128/JCM.43.5.2080-2091.2005 15872226 PMC1153734

[35] Connerton PL, Loc CCM, Swift C, Dillon E, Scott A, Rees CED, Longitudinal study of Campylobacter jejuni bacteriophages and their hosts from broiler chickens. Appl Environ Microbiol. 2004;70:3877–83. 10.1128/AEM.70.7.3877-3883.2004 15240258 PMC444807

[36] Colles FM, Jones K, Harding RM, Maiden MC. Genetic diversity of Campylobacter jejuni isolates from farm animals and the farm environment. Appl Environ Microbiol. 2003;69:7409–13. 10.1128/AEM.69.12.7409-7413.2003 14660392 PMC309911

[37] Manning G, Dowson CG, Bagnall MC, Ahmed IH, West M, Newell DG. Multilocus sequence typing for comparison of veterinary and human isolates of Campylobacter jejuni. Appl Environ Microbiol. 2003;69:6370–9. 10.1128/AEM.69.11.6370-6379.2003 14602588 PMC262249

[38] Rosef O, Rettedal G, Lageide L. Thermophilic campylobacters in surface water: a potential risk of campylobacteriosis. Int J Environ Health Res. 2001;11:321–7. 10.1080/09603120120081791 11798419

[39] Friedman CR, Hoekstra RM, Samuel M, Marcus R, Bender J, Shiferaw B, Risk factors for sporadic Campylobacter infection in the United States: a case-control study in FoodNet sites. Clin Infect Dis. 2004;38(Suppl 3):S285–96. 10.1086/381598 15095201

